# Clinical performance of the near-infrared imaging system VistaCam iX Proxi for detection of approximal enamel lesions

**DOI:** 10.1038/bdjopen.2017.12

**Published:** 2017-06-30

**Authors:** Anahita Jablonski-Momeni, Boris Jablonski, Nikola Lippe

**Affiliations:** 1 Department of Pediatric and Community Dentistry, Dental School, Philipps-University of Marburg, Marburg, Germany; 2 Dental Practice, Lollar, Germany; 3 Department of Periodontology, Dental School, Philipps-University of Marburg, Marburg, Germany

## Abstract

**Objectives/Aims:**

Apart from the visual detection of caries, X-rays can be taken for detection of approximal lesions. The Proxi head of VistaCam iX intraoral camera system uses near-infrared light (NIR) to enable caries detection in approximal surfaces. The aim of this study was to evaluate the performance of the NIR for the detection of approximal enamel lesions by comparison with radiographic findings.

**Materials and methods:**

One hundred ninety-three approximal surfaces from 18 patients were examined visually and using digital radiographs for presence or absence of enamel lesions. Then digital images of each surface were produced using the near-infrared light. Correlation between methods was assessed using Spearman’s rank correlation coefficient (*r*_s_). Agreement between radiographic and NIR findings was calculated using the kappa coefficient. McNemar’s test was used to analyse differences between the radiographic and NIR findings (*α*=0.05).

**Results:**

Moderate correlation was found between all detection methods (*r*_s_=0.33–0.50, *P*<0.0001). Agreement between the radiographic and NIR findings was moderate (*κ*=0.50, 95% CI=0.37–0.62) for the distinction between sound surfaces and enamel caries. No significant differences were found between the findings (*P*=0.07).

**Conclusion:**

Radiographs and NIR were found to be comparable for the detection of enamel lesions in permanent teeth.

## Introduction

The possibilities for accurate caries detection have considerably improved in recent years. In particular, the early detection of initial, non-cavitated carious changes is given a high priority, since preventive and remineralising procedures are well established in modern dentistry and help minimise the need for invasive, often costly restorative treatments when caries is detected early on.

Typically, teeth are first examined by means of a visual inspection in which the clinical assessment of the approximal surfaces is often more difficult because these spaces are harder to access. Bitewing radiographs make it possible to detect approximal lesions that are clinically either undetectable or only partially visible, and provide information on their extension to the pulp. In the case of initial lesions, however, these images are not sufficiently conclusive for detection. The extension of initial caries is often underestimated in bitewing radiographs.^1,2^

Generally, digital bitewing radiographs require potentially lower doses of ionising radiation. Still, there are cases where regularly taking bitewing radiographs is not subject to an indication, e.g., for patients with a low caries risk, or pregnant women. But, it still remains a major task to identify those subjects prone to faster lesion progression in order to use radiography at the most appropriate time.^3^ Several procedures have been developed that do not require radiographs and are used to detect approximal lesions. Some of these existing systems are fibre optic transillumination, laser fluorescence (e.g., DIAGNOdent Pen, KaVo, Biberach, Germany) and the Canary System (Quantum Dental Technologies Inc., Toronto, Canada). In recent years, the focus has returned to the use of light in the infrared or near-infrared (NIR) range^4^ and new instruments were launched on the market. Some of these were, for instance, the DIAGNOcam (KaVo, Biberach, Germany) and the Proxi interchangeable head for the VistaCam iX intraoral camera system (Dürr Dental, Bietigheim-Bissingen, Germany). This interchangeable head was developed as a supplement to the VistaCam iX and the VistaCam iX HD intraoral camera. Two infrared LEDs (850 nm wavelength) are installed in the optical system (6 mW optical output power, dimension of the light spot 7 mm×9 mm) and illuminate the respective mesial and distal enamel area of two adjacent teeth, creating an image by actuating the capture ring. The reflected light is detected by the optical system and is evaluated as a black and white image by the DBSWIN or VistaSoft imaging programme.

The principle here is that NIR light incident on the tooth penetrates through the more transparent enamel and is strongly scattered by enamel lesions and the dentine. The backscattered light causes those areas to appear whiter, compared to sound enamel.^5,6^ The lower the translucency, the higher is the reflection of the infrared light and the brighter the structure. Sound enamel appears dark and approximal caries appears bright. Dentine appears bright due to its low translucency. With regard to the wavelength, some authors described that the near-infrared region between 1,300 and 1,700-nm offers the greatest potential for new optical imaging modalities due to the weak scattering and absorption in sound dental hard tissues.^7^ It could be shown that the transillumination of a tooth using near-infrared light at a wavelength of 1,310 nm leads to a significant contrast between carious and sound enamel, which is well suited for the detection and imaging of interproximal caries lesions.^8^

But, the influence of water in and on the enamel has to be considered as well as the different requirements of an intraoral camera to an imaging sensor. This is why here a wavelength of 850 nm provides the best overall result and diagnostic aid for the examiner.

At present, there are no published studies on the use of the recently launched Proxi head for the intraoral camera system VistaCam iX. Hence the aim of this clinical study was to evaluate the ability of the VistaCam Proxi (NIR) to detect proximal enamel lesions compared to radiographic findings.

## Subjects and methods

The study was independently reviewed and approved by the Ethics Committee of the Medical Faculty, Marburg, Germany (approval number 140/14). The study was performed in accordance with the ethical standards as laid down in the 1964 Declaration of Helsinki and its later amendments. The study was registered at The Coordination Centre for Clinical Trials of Philipps-University Marburg, Germany (project number 1054).

Prior to the study, a power calculation was performed (G*Power 3.1.9).^9^ In expectation of a medium correlation, *α*=0.05 and a power of 0.9, a sample size of 92 surfaces was calculated. A dropout rate of 15% was added to the required sample size. Thus, at least 106 surfaces would have to be included in the study.

No published data were available on the prevalence of approximal lesions in the age group under examination. For the whole country, the mean number of teeth with active initial lesions is 0.7 and for inactive lesions 0.8, respectively, without distinction between the tooth surfaces.^10^

The fluoride concentration in the region’s tap water has been constant for many years and does not exceed 0.25 mg F/l.

The patients were selected sequentially from regular dental screenings. Subjects were at least 18 years of age and gave their informed, written consent prior to their inclusion in the study. Further criteria for inclusion were persons with an indication justifying dental radiographs or for whom radiographs were already available that were not older than 3 months. Pregnant women, minors and patients with more than 12-capped posterior teeth or no posterior teeth were excluded.

This clinical study examined 18 subjects, nine of them were male and nine female. The average age was 29.5 years (18.5–45.8 years). They were examined between February and April 2015 in a dental office.

One examiner was a dentist with a background in cariology, and experienced in the use of ICDAS (International Caries Detection and Assessment System) and caries detection devices. Two further dentists were involved who were experienced in the use of ICDAS theoretically and practically based on their dental educational background and were trained and calibrated within their dental curriculum.^11,12^ Prior to the clinical study, all examiners were trained in the use of the NIR technology based on the manufacturer’s instructions.

All participating examiners used radiographs on a daily basis, nevertheless the classification of proximal caries on bitewing radiographs were discussed prior to the study using bitewing radiographs not included in the study.

For the main study, all findings (visual, radiographs and NIR) were based on consensus decisions. The radiographs and the NIR images were not assessed at the same time. Moreover, the NIR images were classified independently from the clinical and radiographical examinations.

For the visual examinations, a mirror and a ball-ended probe were used after the teeth had been cleaned (using a rotating brush with a prophylactic paste and dental floss afterwards). Teeth were dried with compressed air using a 3-in-1 syringe and isolated with cotton rolls. The visual examination was performed under optimal light conditions based on the criteria of the ICDAS for mesial and distal surfaces are as follows:^13^

*Sound tooth surface—Code 0*: No evidence of caries (either no or questionable change in enamel translucency after 5 s air drying.

*First visual change in enamel—Code 1*: When seen wet, there is no evidence of any change in colour attributable to carious activity, but after prolonged air drying a carious opacity (white or brown lesion) is visible that is not consistent with the clinical appearance of sound enamel. Seen from the buccal or lingual surface.

*Distinct visual change in enamel when viewed wet—Code 2*: Carious opacity or discoloration (white or brown lesion) that is not consistent with the clinical appearance of sound enamel. The lesion is still visible when dry. This lesion may be seen directly when viewed from the buccal or lingual direction. In addition, when viewed from the occlusal direction, this opacity or discoloration may be seen as a shadow confined to the enamel, seen through the marginal ridge.

No further distinction between active and inactive lesions was made for the ICDAS 1 and 2 codes. Approximal surfaces with ICDAS codes higher than 2, or enamel changes not due to caries, were not included in the study. For further analyses, ICDAS 1 and 2 codes were merged (ICDAS initial).^14^

The teeth were separated prior to the visual examination using a dental wedge, but without additional separation of the teeth using orthodontic separation elastics. After the visual classification was performed, the digital radiographs of the corresponding approximal surfaces were examined. The radiographs were taken with a digital X-ray machine (Heliodent DS, Sirona Bensheim, Germany) using a CCD intraoral sensor and a sensor holder. The radiographs were adjudged by consensus by the examiners according to the following classification:^14^

D0=No radiolucency;

D1=Radiolucency in the outer ½ of the enamel;

D2=Radiolucency in the inner ½ of the enamel±EDJ (enamel–dentine junction).

For the analyses, distinction was made between D0 and D1+D2 (enamel lesions).

### Use of Proxi interchangeable head (NIR)

Images were taken from all teeth included in the study using the Proxi interchangeable head with the corresponding positioning holder. A disposable hygienic protective cover was used on each patient for hygienic reasons. The optical system was placed on the row of teeth above the approximal area and images were taken according to the manufacturer’s instructions using the DBSWIN software (Version 5.9.0). The depth of the lesions on the images was assessed independently of the visual and radiographic findings. Since there were no published classifications in the regard, a classification was constructed according to the manufacturer’s information and defined as follows:

0=no signs of changes in enamel;

NIR 1=wide bright strip or wedge-shaped structures within the dark translucent enamel. The lesion may reach the enamel–dentine junction;

NIR 2=wide bright strip or wedge-shaped structures, which seem to cross the enamel–dentine junction.

For the statistical evaluation, the NIR 1 and NIR 2 were categorised as lesions into enamel.

The treatment of the carious lesions was performed on the basis of the previous dental findings independently of the IR findings. Since it was a question of non-cavitated initial lesions, no operative treatment was given to approximal surfaces, but preventive action taken instead (as a rule, professional tooth cleaning, local fluoridation and oral hygiene instructions) and the patients were scheduled for regular recalls.

**Figure 1:**
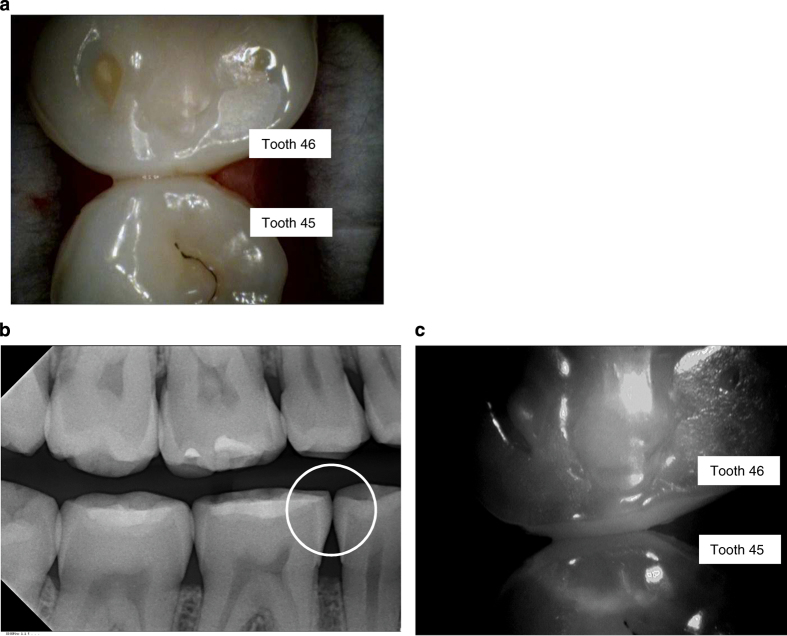
Examples of images using the VistaCam iX Proxi head. In some NIR images, a halo can be seen in the tooth, which is presumably due to the fact that light gets reflected from the round glass on the inner lens of the camera. The reflection can only be seen on white background. This lens has been replaced in the new commercially available device by a specifically coated lens that does now longer cause this irritation. (**a**) Image of teeth with the intraoral camera (45 distal, 46 mesial). (**b**) Corresponding bitewing radiograph (no radiolucency seen in 45 distal/46 mesial). (**c**) Corresponding NIR image.

**Figure 2:**
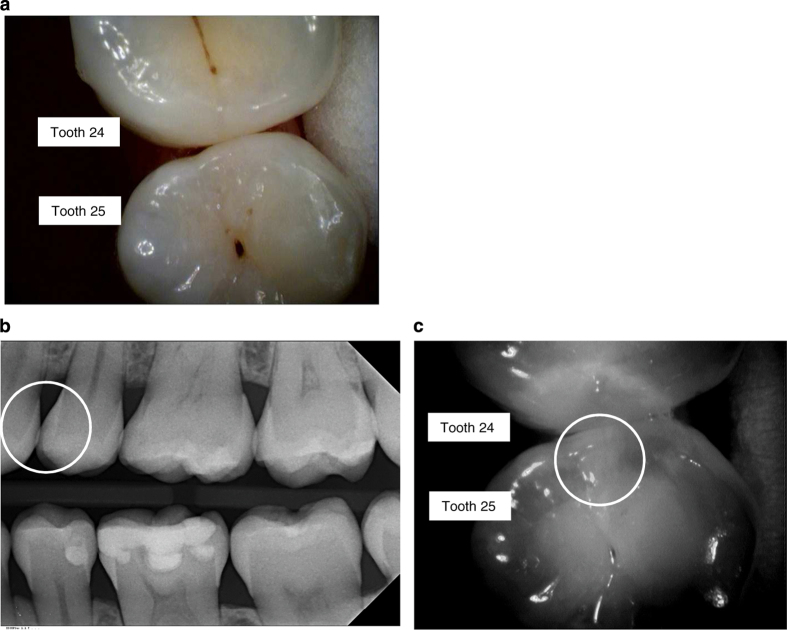
Examples of images using the VistaCam iX Proxi head. In some NIR images, a halo can be seen in the tooth, which is presumably due to the fact that light gets reflected from the round glass on the inner lens of the camera. The reflection can only be seen on white background. This lens has been replaced in the new commercially available device by a specifically coated lens that does now longer cause this irritation. (**a**) Image of teeth with the intraoral camera (24 distal, 25 mesial). (**b**) Corresponding bitewing radiograph (D1 lesion seen in 25 mesial). (**c**) Corresponding NIR image. The circle indicates the area that is associated with demineralisation of the enamel.

**Figure 3:**
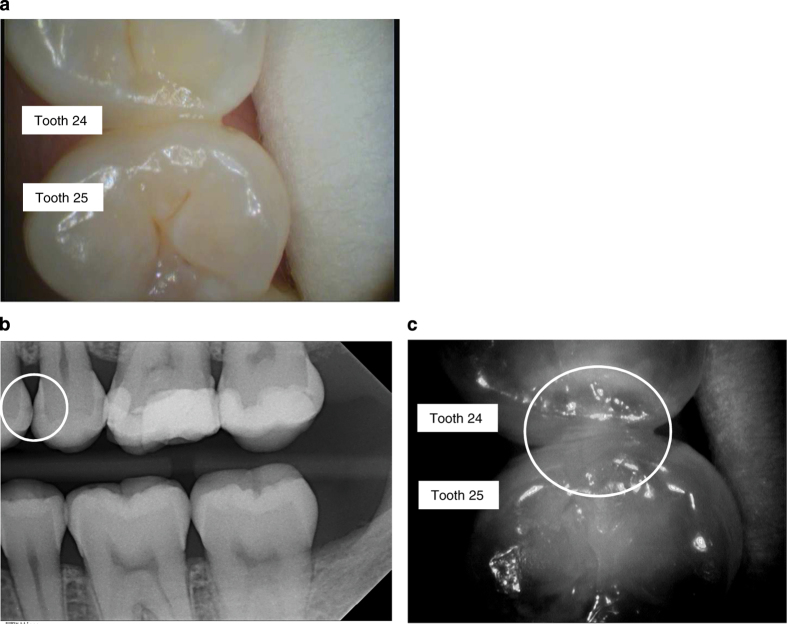
Examples of images using the VistaCam iX Proxi head. (**a**) Image of teeth with the intraoral camera (24 distal, 25 mesial). (**b**) Corresponding bitewing radiograph (D2 lesion seen in 24 distal, D3 lesion seen in 25 mesial, this surface was not included in the study). (**c**) Corresponding NIR image. The circle indicates the area that is associated with demineralisation of the enamel.

**Figure 4:**
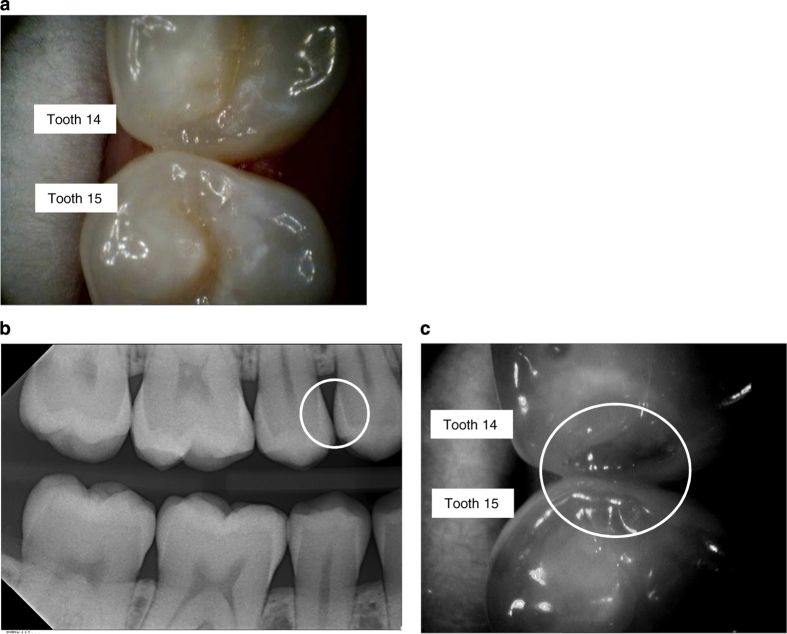
Examples of images using the VistaCam iX Proxi head. (**a**) Image of teeth with the intraoral camera (14 distal, 15 mesial). (**b**) Corresponding bitewing radiograph (no radiolucency seen in 14 distal/15 mesial). (**c**) Corresponding NIR image. The dark area shows that the crystal structure is intact in this area. The slightly more bright impression left to this is caused by the radius in which the surface is bent.

Example of clinical, radiographic and NIR images are presented in Figures 1,Figures 2,Figures 3,Figures 4.

### Statistical analysis

Statistical analysis was performed using the MedCalc statistical software, Version 16.4.2 (Ostend, Belgium). The distribution of the findings (visual examination, radiographic findings and NIR scores) was represented using cross tabulation and analysed using the chi-square test (*χ*^2^). Correlation between all methods was assessed using Spearman’s rank correlation coefficient (*r*_s_). Agreement between radiographic and NIR findings was calculated using the kappa coefficient. McNemar’s test was used to analyse differences between the findings. The significance level was set at *α*=0.05.

## Results

A total of 193 approximal surfaces from 161 posterior teeth were included in the study (95 surfaces of molars, 98 surfaces of premolars). On the basis of the visual criteria, 74 surfaces (38.4%) were sound, whereas 78 surfaces (40.4%) were categorised as ICDAS 1 and 41 surfaces (21.2%) as ICDAS 2.

Sixty-three surfaces (32.6%) showed radiographical signs of enamel lesions (41 surfaces D1, 22 surfaces D2) and 130 surfaces (67.4%) were without radiographic signs of caries on proximal surfaces (D0). The distribution of the findings (merged codes) is presented in Tables 1–3. There was a significant positive correlation between all methods (*χ*^2^-test, *P*<0.0001).

It was not possible to evaluate 8 of 193 images with the NIR (4.1%) mainly due to strong reflection of light. These were four times on premolars and four times on molars. On the corresponding bitewing radiographs, no caries were found for four of these images, while caries in the first half of the enamel was defined on three approximal surfaces and caries up to the enamel–dentine junction on one surface. On four NIR images, the whitish change seemed to go beyond the enamel–dentine junction (NIR code 2). These were diagnosed as without caries in one radiograph and as caries up to the enamel–dentine junction in three radiographical images.

Forty-four (69.8%) of those 63 surfaces with radiographical signs of enamel lesions were assessed on NIR images (Table 3). From the 130 radiographical sound surfaces, a total of 28 surfaces (21.5%) showed signs of enamel lesions on the NIR images (Table 3).

The agreement between the radiographic and NIR findings was moderate (*κ*=0.50, 95% CI=0.37–0.62) for the distinction between sound surfaces and enamel caries. McNemar’s test showed no significant differences between the findings (*P*=0.07). Agreement between visual findings and both imaging methods was fair: ICDAS/radiography: *κ*=0.28, 95% CI=0.17–0.39 (*P*<0.0001); ICDAS/NIR: *κ*=0.37, 95% CI=0.25–0.49 (*P*<0.0001).

Rank correlation between all methods was moderate (Table 4) and significantly positive (*P*<0.0001, two-tailed).

## Discussion

This study investigates for the first time the suitability of the Proxi head on the VistaCam iX intraoral camera system for detecting approximal lesions, comparing the results with the findings of radiographic images. The Proxi head was developed primarily for the detection of enamel lesions. However, no differentiation in the enamel was carried out in the images produced here, since a clear detachment of the enamel was only possible to a limited extent. The images were produced from the occlusal site of the teeth and thus the images are not necessarily congruent with bitewing radiographs. A classification of caries in two enamel halves rarely influences a therapy decision, however, because preventive, non-operative care is generally given preference in this initial stage. Operative care of lesions with the aim of tooth preservation is only necessary when the caries reaches moderate or extensive stages, depending on the individual caries risk.^15^

The visual detection of approximal caries is often challenging, since these surfaces are usually hidden by adjacent teeth, thus preventing a direct view of the tooth surface to be assessed. Exceptions here are teeth that are easy to access because the adjacent tooth is missing or not yet erupted. In order to better represent approximal surfaces, teeth can be separated using orthodontic separation elastics.^16^ Usually, enough space is created within a few days for a better visual and, if needed, tactile examination of the tooth surfaces. For organisational reasons, however, the present study did not avail itself of long-term separation of teeth, only separating them slightly with a dental wedge shortly before the visual examination. The correlation that the study found between visual findings and radiographs was lower than the results of *in vitro* examinations, in which the approximal spaces can be seen directly.^17^ In a recently published study using the device KaVo DIAGNOcam for approximal caries detection, no separation of the teeth was reported.^18^ The authors report a high concordance between the transillumination method and clinical examination.

In the daily clinical situation, radiography is the most widespread lesion detection aid, particularly with respect to otherwise invisible or poorly visible approximal areas.^3^ Nevertheless, radiography always carries risks associated with the use of ionising radiation. Of course, the radiation exposure from a digital X-ray machine is many times less than that from a conventional radiographic method.^19^ Nonetheless, noninvasive methods of detection are always of benefit as an alternative to radiographs. As a basic principle, the frequency of bitewing radiographs should be adjusted to the caries risk of the individual patient.^20,21^ Of the teeth included in the study, 67.4% had no radiographic signs of approximal caries; hence the population can be defined being at low caries risk.

In previously published studies, a higher contrast between sound enamel and initial lesions was found in NIR images compared to radiography, which indicates a higher sensitivity of NIR in detection of caries lesions.^8,22^ In a clinical study it could be shown that 97% of the lesions that appeared on bitewing radiographs were seen in the NIR images (1,310 nm wavelength).^23^ Many areas on the teeth that appeared to be demineralised in the NIR images did not show up on the bitewing radiographs. This suggests that NIR may be more sensitive than bitewing radiographs for imaging early lesions. However, the authors discussed that confirming this clinically is not straightforward since such small lesions do not require restoration and since the teeth are not extracted, there is no suitable gold standard for comparison.^23^ The results of our study revealed that 21.5% of the radiographical sound surfaces showed signs of enamel lesions on the NIR images (Table 3). Still, there is a lack of a valid gold standard since no tooth was planned for extraction and for further histological validation. For ethical reasons, it was not acceptable to follow the visual detection of suspected enamel caries and open lesions to corroborate the clinical assessment. Here the reference was based on radiographic examinations without any assessment of the lesion depth after opening. Visual examination cannot be used as a gold standard and problems may occur since even with appropriate training and calibration, there is always the risk of subjective interpretation of lesion extent or severity. However, with the visual assessment, more proximal lesions were detected than with the radiographic and NIR assessments (Tables 1 and 2) and hence the visual detection seems to be far superior to the other methods used in this study. In another study using histology as gold standard high-sensitivity values for the use of ICDAS 1–3 scores on proximal surfaces were reported.^24^ The results were statistically higher than the results of conventional and digital radiography. In contrast, the authors reported higher specificity values for the radiography than for the visual scoring system. The authors concluded that visual examination using the ICDAS criteria is a promising method for the detection of initial proximal lesions and facilitates early diagnosis and the adoption of preventive measures. The data presented in our clinical study support this. Since initial lesions in proximal surfaces cannot be presented properly by means of photographs, the use of imaging systems without radiation may be a promising way to objectify such lesions and can be used for assessment and monitoring purposes.

It might be argued that the lack of a histological gold standard could lead to less valid results. Also, this may also lead to the presumption that there is potential for false positives since there was no confirmation that the lesions identified with the NIR only were actually lesions. One possible approach could be follow-up appointments for reassessment of the teeth and monitoring the surfaces when no operative treatment is required,^25^ depending on the individual caries risk. It is important to question what impact this would have clinically. The relevance of the differentiation between entirely sound hard tooth tissue and incipient lesions is often of secondary importance to the therapeutic consequence, insofar as the individual caries risk of a patient does not make operative treatment of lesions advisable.^25^ The clinical outcome would therefore consist in taking preventive care on a sound site, which would still be of benefit to a high-risk patient.^26^

When teeth are scheduled for extraction, for example due to orthodontic reasons it is possible to use histology as a gold standard after extraction. Nevertheless it should be taken into account that it usually leads to a selection bias when teeth are planned for extraction and not all ranges of dental caries can be included in the study.

If we look at the images in this study, there is a specular reflection present in some of the images. The device has to be positioned orthogonal to the enamels surface. The more the angle at a certain position differs from this, the more likely it is to get a full/specular reflection. The bright reflections can also be caused by wet surfaces. For the diagnosis, this is not relevant because the examiner can easily differentiate between these extremely bright spots and the white/greyish areas that are typical for reflections caused by a change of the crystal structure. A change of the crystal structure causes a more diffuse reflection. A specular reflection can also be caused by a filling, e.g., amalgam or a ceramic inlay in the tooth. By slightly moving the device before taking an acquisition, it can also easily be seen that the specular reflections come and go while the ones caused by changes in the crystal structure will still be visible. In some NIR images (Figures 1 and 2), a halo can be seen in the tooth which is presumably due to the fact that light gets reflected from the round glass on the inner lens of the camera. The reflection can only be seen on white background. This lens has been replaced in the new commercially available device by a specifically coated lens that does now longer cause this irritation.

The device we used based on the near-infrared light technology was designed for enamel lesions. This differs from the DIAGNOcam, although using a similar wavelength of light. Initial clinical studies with the DIAGNOcam showed a high level of diagnostic accuracy for the detection of approximal dentine caries in the area of the enamel–dentine junction.^27^ However, only dentine lesions were included in that study and the results are thus not comparable to our data.

In occlusal lesions, high contrast between sound and demineralised areas with NIR could be assessed.^28^ It was concluded that NIR offers significant advantages over conventional visual, tactile and radiographic caries detection methods for detection of early stages of lesions in occlusal pits and fissures, which cannot be detected by X-rays due to the overlapping topography of the crown of the tooth. Moreover, demineralisation can be easily differentiated from stains, pigmentation and hypomineralization (fluorosis). The high transparency of the enamel enables imaging at greater depth for the detection of subsurface decay hidden under the enamel.^28^ With NIR, high-rank correlation with histology was obtained, as well as a sensitivity of >99% and a specificity of 87.5% for occlusal enamel lesions.^29^ In addition, it was shown that stain and pigmentation did not show a significance interference with the NIR measurements, which is an advantage to visual inspection when teeth are not cleaned properly.^29^

## Conclusion

The results of our study showed that the Proxi head of the intraoral camera VistaCam iX can provide findings comparable to those of radiographs for non-cavitated lesions. The pulp cannot be represented with this device, however. Hence, radiographs are still preferable for indications of this nature. When linked directly to a patient database, digital documentation and monitoring of lesions are possible, without exposure to radiation usual with bitewing radiographs. Using this camera can be recommended especially for patients with an enhanced caries risk, but also for children and pregnant women. Hence, further research should focus on these groups. Also, the camera has additional heads which can be used to take not only regular intraoral images, but also images of occlusal and smooth surfaces for caries detection and dental plaque (fluorescence head).^30,31,32^

## Figures and Tables

**Table 1:** Cross tabulation of visual and radiographic findings

*Digital radiography*	*Visual examination*		*N Total (%)*
	*Sound*	*ICDAS initial (ICDAS 1+2)*	
No radiolucency (D0)	65	65	130 (67.4%)
Radiolucency in enamel (D1+D2)	9	54	63 (32.6%)
N Total (%)	74 (38.4%)	119 (61.6%)	193 (100%)

**Table 2:** Cross tabulation of visual and near-infrared findings

*Near-infrared light*	*Visual examination*		*N Total (%)*
	*Sound*	*ICDAS initial (ICDAS 1+2)*	
No signs of changes in enamel (NIR 0)	62	51	113 (58.5%)
Wide bright strip or wedge-shaped structures within the dark translucent enamel (NIR 1+ NIR 2)	10	62	72 (37.3%)
Not assessable	2	6	8 (4.1%)
N Total (%)	74 (38.4%)	119 (61.6%)	193 (100%)

**Table 3:** Cross tabulation of radiographical and near-infrared findings

*Near-infrared light*	*Digital radiography*		*N Total (%)*
	*No radiolucency (D0)*	*Radiolucency in enamel (D1+D2)*	
No signs of changes in enamel (NIR 0)	98	15	113 (58.5%)
Wide bright strip or wedge-shaped structures within the dark translucent enamel (NIR 1+ NIR 2)	28	44	72 (37.3%)
Not assessable	4	4	8 (4.1%)
N Total (%)	130 (67.4%)	63 (32.6%)	193 (100%)

**Table 4:** Spearman’s rank correlation coefficients (*r*_s_) and 95% CI between all methods

	*r*_*s*_ *(95% CI)*	
	*Digital radiography*	*Near-infrared light*
Visual examination	0.33 (0.20–0.46)	0.41 (0.28–0.52)
Digital radiography	—	0.50 (0.38–0.60)

All correlations are significantly positive: *P*<0.0001, two-tailed testing.
